# Policy analysis of socio-cultural determinants of salt, sugar and fat consumption in Iran

**DOI:** 10.1186/s40795-022-00518-7

**Published:** 2022-03-25

**Authors:** Mohammad Amerzadeh, Amirhossein Takian, Hamed Pouraram, Ali Akbari Sari, Afshin Ostovar

**Affiliations:** 1 Social Determinants of Health Research Center, Research Institute for Prevention of Non-Communicable Diseases, Qazvin university of Medical Sciences, Qazvin, Iran; 2 Department of Health Management, Policy & Economics, School of Public Health, Tehran University of Medical Sciences (TUMS), Tehran, Iran; 3 Department of Global Health and Public Policy, School of Public Health, Tehran University of Medical Sciences (TUMS), Tehran, Iran; 4 Heath Equity Research Center (HERC) – TUMS, Tehran, Iran; 5 Department of Community Nutrition, School of Nutritional Sciences and Dietetics, Tehran University of Medical Sciences (TUMS), Tehran, Iran; 6 Center for NCD Prevention and Management, Ministry of Health and Medical Education, Tehran, Iran; 7 Osteoporosis Research Center, Endocrinology & Metabolism Research Institute, Tehran University of Medical Sciences (TUMS), Tehran, Iran

**Keywords:** Socio-cultural determinants, NCDs, Policy analysis, Fat, Sugar, Salt, Iran

## Abstract

**Background:**

Noncommunicable diseases (NCDs) are the first reason for death worldwide, in which poor diet is the leading risk factor. It is estimated that 20% of all death is related to food. The Unhealthy diet includes many foods with excessive salt, sugar and fat. This paper reports a national study on the socio-cultural determinants affecting salt, sugar and fat consumption in Iran.

**Methods:**

This is a qualitative study. We conducted semi-structured interviews with 30 various purposefully identified key stakeholders to collect data from December 2018 until August 2019 in Iran.

**Results:**

We identified socio-cultural determents of salt, fat and sugar consumption as follows: Inadequate structure of traditional medicine and people’s desire for traditional foods, low health literacy, the global trend of nutritional transition and its impact on Iranian society, The progressive decline of people’s trust in NGOs, and Inappropriate media management. Worse still, the global trend of nutritional transition and people’s tendency towards fast foods, unhealthy diet and junk foods, partially due to establishing children’s taste mainly with salty, high-fat and sweet foods, has jeopardized their desire to eat healthily during adulthood.

**Conclusion:**

Reducing salt, fat and sugar consumption is problematic in Iran, mainly due to multi-dimensional socio-cultural determinants. In line with sustainable development goal (SDG) 3.4 to reduce 30% of premature death due to NCDs and related risk factors by 2030 in Iran, various stakeholders from multiple sectors need to initiate coherent series of interventions to alter people’s approach to select food so that they may reduce the consumption of foods with excessive salt, fat and sugar.

**Supplementary Information:**

The online version contains supplementary material available at 10.1186/s40795-022-00518-7.

## Background

Noncommunicable diseases (NCDs) are the leading causes of death globally [1]. Four significant NCDs, including cardiovascular and respiratory diseases, cancers, and diabetes, cause over 74% of annual death worldwide (42 million) [2]. Approximately 85% of premature deaths due to NCDs occur in low and middle-income countries (LMICs). Major risk factors of NCDs are tobacco use, physical inactivity, the harmful use of alcohol and unhealthy diets [2, 3]. Poor diet is the leading risk factor for NCDs and related death worldwide [4]. Improvement of diet can potentially prevent 20% of deaths globally [5].

Based on the WHO’s recommendations, balance of energy intake is important, in a way that total fat intake should not exceed 30% of total energy, while saturated fats and trans-fats intake should be less than 10 and 1% of the entire energy intake, respectively [6–8]. Free sugar intake should be limited to less than 10% of total energy, and more reduction to less than 5% of the entire energy intake is recommended for extra health benefits [9]. Salt intake should also be less than 5 g per day to prevent hypertension and decrease heart disease and stroke risks [10]. Further, evidence shows that equal access to various micronutrient-rich foods, e.g. fruits, vegetables, legumes, pulses, and nuts is a global challenge [11]. Whereas, unhealthy foods with a high amount of salt, sugar, saturated and trans fats have become more affordable and available worldwide [11]. Let alone, demand for meat, dairy products, sugar-sweetened drinks, and processed and ultra-processed foods has risen worldwide [11].

The World Health Assembly (WHA) has also approved nine voluntary targets to reduce NCDs’ behavioural and physiological risk factors. They include a target to reduce mean population salt intake by 30% by 2030, a 25% relative reduction in the overall mortality from cardiovascular diseases, cancer, diabetes, or chronic respiratory diseases and halting the rise in diabetes and obesity [12]. WHO has also identified a set of interventions, so-called “best buys”, which are highly cost-effective, feasible and appropriate to the context of LMICs [13]. In line with WHO Global Action Plan, Iran developed “The National Action Plan for Prevention and Control of NCDs and the related risk factors, 2015–2025”, revised to cover the targets until 2030. It contains salt intake reduction by 30% until 2030, and zero trans-fatty acids in food & oily products by 2020 [14].

Various factors determine healthy eating, in which sociocultural factors, including cultural norms, social pressures, social class, social networks, and race/ethnicity, play important roles [15]. Researchers have also pointed out that cultural, social, environmental, and psychological forces significantly affect health behaviour and behavioural change. Therefore, personalized cognitive-behavioural and culturally and socioeconomically sensitive strategies help change behavior [15–17]. NCDs cause 83% of premature death, and dietary risks are among the first line of NCDs’ risk factors in the country [2, 18, 19]. We earlier reported existing policies to reduce sugar, salt, and fat consumption in Iran [20]. This paper reports the findings of a national study on the sociocultural factors, mechanisms, and policies to reduce salt, sugar and fat consumption in Iran. Our findings will assist, we envisage, the way to achieve the goals of national action plans for prevention and control of NCDs, particularly SDG 3.4 to 30% reduction of premature death because of NCDs by 2030 in Iran, and perhaps similar settings.

## Methods

### Study design & data collection

This qualitative study aims to analyze socio-cultural determinants affecting salt, sugar, and fat consumption in Iran. We collected data through 30 semi-structured, in-depth and face-to-face interviews with stakeholders, experts and key informants who had experience and knowledge in health promotion and planning, policymaking and management of different healthcare levels in the health system. Through literature review and in line with the Government Healthy Food Environment Policy Index (Food-EPI) monitoring tool [21], we developed a generic interview guide for data collection. The guide has two components: policies and infrastructures (Appendix 1). We used the health policy triangle framework for deductive analysis [22]. The Interviews were recorded digitally and transcribed verbatim. We provided the interviewees with an information sheet, reassured them about anonymity, freedom to withdraw and confidentiality, explained the purpose of the study and requested them to read and approve the informed consent form.” The Ethical Committee of Tehran University of Medical Sciences (TUMS) approved this study (the ethical code: IR.TUMS.REC.1397.193). Interviews lasted about 30–90 min and were conducted from December 2018 until August 2019 in Tehran- Iran. The first author (MA) conducted all interviews, except for the first one, in which the corresponding author (AT) interviewed in the presence of MA. Notes were made during the interviews, and the place, date, time, and other significant issues were all recorded.

### Setting and sampling

We used a purposeful sampling approach to select our participants from four groups: academics, healthcare services providers, national policymakers, and regulatory organizations, with maximum variation. Initially, we purposefully selected key informants and relevant organizations and institutions by reviewing related documents and reports. We then identified and added other key stakeholders through the key informants identified in the previous step (snowball method) and continued the interviews until we reached saturation. We tried to choose organizations that represented key actors related to diet and NCDs. Table 1 summarizes the list of interviewees.

**Table 1:** The list of interviewees and their scope

Scope of Interviewees	Number
Department of Community Nutrition in Ministry of Health and Medical Education (MOHME)	2
NCDs Management Office- The MOHME	1
National Committee for Prevention and Control of NCDs	2
Schools of Nutrition affiliated with Universities of Medical Sciences	2
Deputy for Health- The MOHME	1
Health Education and Promotion Office - The MOHME	1
The Iranian Academy of Medical Sciences	1
Food and Drug Administration (FDA)	3
Supreme Council of Health and Food Security	2
Primary Healthcare Network Office- The MOHME	1
Ministry of Agriculture	1
Ministry of Industry, Mine and Trade	1
Ministry of Education	1
Deputy of Health at the Universities of Medical Sciences	2
Municipalities	2
Standard Organization of Iran	1
Ministry of Economic Affairs and Finance	1
Islamic Republic of Iran Broadcasting (IRIB)	1
Deputy of Curative Affairs- The MOHME	1
WHO Country Representative in Iran	1
Planning and Budget Organization	1
Non-Communicable Diseases Research Center	1

### Data analysis

We conducted qualitative content analysis and utilized a mixed deductive and inductive approach for data analysis [23, 24]. We collected and analyzed the data simultaneously. At the end of each interview, the first author transcribed them verbatim and read them several times for familiarization. We classified qualitative data into three levels in an inductive approach: open coding, creating categories, and abstraction. All authors took part in the coding tree interpretation under the supervision of the corresponding author. The first author carried out the open coding by identifying and naming the phenomena described in the text. In this regard, we extracted meaning units based on the study’s aims, and then we labelled each meaning unit called a code. MAXQDA software (version 11) was used to help with data storage and categorization. Following the open coding, we categorized the codes into various groups and compared the codes with each other, changed and moved them constantly to display the message of the interviews. The first and corresponding authors carried out the categorization process. Nonetheless, all authors revised and approved the entire steps. In the end, to increase the credibility, we sent the transcripts, categories, subcategories and codes to some selected interviewees and sought their approval to make sure the accuracy of the interpretations [25, 26].

## Results

Table 2 summarizes the socio-cultural determinants of salt, sugar and fat consumption in Iran, as well as the identified cultural barriers.

**Table 2:** Cultural barriers on standard consuption of salt, sugar and fat in Iran

Theme	Category	Examples of cultural barriers
Cultural factors	Inadequate structure of traditional medicine People’s desire for traditional foods	Our religious leaders used to eat salt with their food More consumption of traditional bread, cheese and dough (yogurt drink) which contain more salt and fat
Individual factors	Low health literacy	Families do not consider the nutritional principles and establish children’s taste mainly with salty, high-fat and sweet foods
Commercial factors	The global trend of nutritional transition and its impact on Iranian society	The tendency for fast foods and junk foods has increased
Social factors	The progressive decline of people’s trust in NGOs	Few NGOs work and assist the government in these fields
Media factors	Inappropriate media management	The potential of these tools seems to be under-utilized, and more unreliable information spread and people do not consider public health recommendations

### Inadequate structure of traditional medicine and people’s desire for traditional foods

Misinterpretation of traditional medicine and inadequate structure and training of some non-scientific principles by some people seem to hurt people’s health. Some people also think traditional products like cheese and bread are healthier. For example, based on some narrations, some people express that some religious leaders used to eat salt with their food or sometimes their dominant diet was bread and salt. Nowadays, physical activity is not the same as it used to be, and transpiration is less:

"*For example, some people express that our religious leaders recommended eating salt before and after the meal, which confuses the amount and the type of salt used. Moreover people use more sea salts instead of iodized salt." (PMN2)*

Further, people are more interested in traditional foods such as traditional bread, cheese and dough (yoghurt drink), which are more compatible with their tastes. Some people think these products are healthier. Nevertheless, in these non-industrial foods, salt control is more complicated, and many salt producers are reluctant to observe salt levels. Besides, monitoring is not enough:

*“Unfortunately, many people prefer traditional foods, believing that they are healthier. One of the social problems is that people dislike low-salt cheese because it is not compatible with their taste.” (PMN9)*

Further, the media aggravates the problem by inviting some incompetent people and non-scientific advertising. For example, people tend to eat sea salt, imagining that sea salt is healthier and has no side effects, such as industrial salt. However, the MOHME could promote iodized salt in the community for many years with lots of effort and decrease many problems of goitre and related disease:

*“Advertisements show that sea salt does not have side effects of industrial salt, while industrial salt is hygienic and has iodine.” (PMN9)*

### Low health literacy

Some interviewees considered the low level of public health literacy as one of the main issues affecting the country’s consumption of sugar, salt, and fat. They believed that increasing public health literacy would reduce health-threatening behaviours, increase awareness, prevent many illnesses, which might lead to a healthier society:

*"Students’ health literacy is very low. Media, universities and schools should promote health literacy.” (HEN18)*

One reason for the low public health literacy is the lack of awareness of families and the lack of effort to increase it. Some interviewees considered the fundamental cause of the problem in their families and believed that families do not observe the nutritional principles and therefore establish children’s tastes wrongly:

*“Our taste is based on a model, and our parents do not spend much time learning, don’t have information, and are not interested in appropriate learning properly. Therefore, the baby’s taste will form wrong. For example, when a mother starts providing supplementary food for her child at the age of six months, she tastes herself, which means she is forming the baby’s taste similar to her taste. This is where you have to intervene based on scientific principles.” (PMN16)*

### The global trend of nutritional transition and its impact on the Iranian society

The dietary pattern has changed globally because of industrialization and time constraints to make healthy foods at home; hence, the tendency for fast foods and junk foods has increased. Besides, although there are many healthy and various foods in the country, these kinds of food might be offered less frequently in restaurants:

*“It’s a global trend. Many people in the world eat hamburgers and pizzas. It highly exposes the kids to these ingredients.” (PMN2)*

*“Unfortunately, restaurants do not pay attention to this variety of food because it is not profitable for them. When people go to the restaurant, they expect to eat foods not available at home. Therefore, tourists’ reports of our country are based on restaurant foods. “(PMN12).*

### The progressive decline of people’s trust in NGOs

In many countries, NGOs play various roles in the society and the health sector by assisting the government in various ways. For example, NGOs help train people about nutrition principles, how to cook food properly, the principles of infant nutrition, the distribution of food during emergencies, school nutrition monitoring, etc. Nonetheless, few NGOs work and assist the government in these fields in Iran. Some interviewees mentioned that most NGOs are government-established NGOs, and people do not trust them because of their poor performance:

*“We are still young in terms of the partnership, even though it has been over 100 years and people’s trust has been ruined somehow.” (HEN11)*

Besides, some interviewees attributed this problem to the overall low social capital and inadequate health-based perspective in society. They pointed out that people’s trust in government agencies and affiliated NGOs have decreased:

*“Regarding NGOs in Iran, our overall social capital is low in society. Social capital has different levels. Our social capital is also disrupted at the first level. We also have problems at the family level.” (PMN10)*

One local policymaker also pointed to the low social capital in the country:

*“In general, since social capital in Iran has decreased, people do not somehow trust the governmental agencies.” (PMN16)*

There are also very few NGOs in the field of salt, sugar and fat control whose work is related to the prevention and training of these problems; NGOs can make people aware and reduce salt, fat and sugar consumption. One reason is that most NGOs in the country deal with treatment, e.g. diabetes, cancer, kidney patients and so on:

*“Most of those who come to us for NGOs are related to treatment. They aim to help patients more. There are very few NGOs in the field of nutrition.” (PMN20)*

### Inappropriate media management

One tool to train people about healthy nutrition is the media and civil society. Regarding the widespread use of social networks and their impact on society, the potential of these tools seems to be underutilized in healthy diet education. Some interviewees believed that with the advent of new media, the government does not promote the culture of its usage, and it is common to push people away with negative advertising:

*" Social networks have grown much faster than the cultural standards of their usage. The culture of using social networks is very low in the country. The media and film industry can be very effective." (HEN25)*

Besides, another socio-cultural problem in recent years is promoting foreign (broadcasted from outside of Iran) media and people’s interest in them. According to some interviewees, the source of a lot of news and information comes from foreign media, which has reduced the impact of domestic media. As a result, less reliable information on health and nutrition can be transferred to the public and more unreliable information spread:

*“How do we want to convey information to people? How popular is the IRIB (Islamic Republic of Iran Broadcasting)? Nowadays, more and more people are watching foreign channels.” (HEN18)*

## Discussion

This study analyzed policymaking regarding the socio-cultural determinants affecting sugar, salt, and fat consumption in Iran, aiming to revise and improve related policies.

Our findings show that the inadequate structure in traditional medicine, training some non-scientific principles by some people, people’s desire for conventional foods, and low health literacy are among socio-cultural determents influencing high sugar, salt, and fat consumption in the country. This problem of traditional foods containing a high level of fatty acid and salt also exists in Vietnam, drawing particular attention [27]. The root of the problem is the lack of families’ awareness and inadequate effort to raise it, which might establish children’s taste mainly with salty, high-fat and sweet foods from the early stages. In another study, which examined the knowledge and attitudes towards salt consumption in people referred to the Health Center in Ardabil (a province in the north-west of Iran), the total score was 7.5 out of 16, which shows a low level of awareness [28]. A similar study emphasizes increasing health-related knowledge, abilities, and skills to bridge the gap between knowledge and dietary practices and to raise health literacy in society [29]. Therefore, more monitoring of traditional medicine and increasing people’s and families’ health literacy are important actions.

Further, our finding reveals a problem with the media and social networks. It seems that the government does not use the potential of social media appropriately and sufficiently to boost healthy diet education. With the advent of the new media, the contextual culture of its usage has not been promoted in Iran. Besides, people’s interest in domestic media and its impact might also decrease. Many untrustworthy channels transfer wrong and unreliable information (infodemics) about sugar, salt, fat intake, and nutrition to the public. At the global level, mass-media awareness campaigns, plus the regulations related to salt content, can avoid 8.5 million deaths worldwide [30]. The problem might be the availability of reliable bodies to monitor the content of cyberspace to ensure the credibility of a source. Another study highlights the importance of social media and its role in improving people’s diet [31].

Our interviewees also expressed that the pattern of nutrition has also changed globally, mainly because of industrialization. People’s tendency towards fast foods, unhealthy foods and junk foods has increased. Although there are many healthy and different foods in the country, they are less offered in restaurants, while most served foods are unhealthy regarding salt, sugar and fat. Another study found that girls in Lahijan (a northern city of Iran) had a high intake of low-value snacks and high-calorie foods [32]. There was also the problem of changing people’s eating habits and eating more fast food and foods containing sugar, salt and fat in Nepal, Pakistan and Bangladesh [33].

Our research endorses other studies that suggest that fast food and new-generation tastes need special attention. One reason for the fast-food tendency is because people have less free time and do not have enough time to cook meals, especially housewives. Another reason is the low price of these foods because of the lack of healthy foods, which might lead people to buy them because of economic problems. Another study about the spread of fast food found different reasons for this issue, including the Perfect Meeting Place, disliking traditional foods, transition in family structure and values, media Influence, peer pressure, marketing strategies, awareness of food production, and lower price [34].

We found another problem with the Iranian diet, which is people’s taste desire for salty, high-fat and sweet foods. Many manufactures use salt in most foods, such as cheese, because of its protective properties, while consumers are also interested in such a taste. Similar to our study, another study refers to Iranian salty food consumption habits and the fact that the taste of salt exists in most traditional Iranian dishes. It also considers manufacturing and offering high salt products in the food industry a threat [31]. Another study recommends appropriate subsidy allocation for healthier food products, such as flour and bread to promote beneficial nutrition in society and better purchasing habits [35]. Therefore, the food culture needs to be changed gradually and use less salt, sugar and fat for different dishes.

### Rigor of study

To the best of our knowledge, this study is the first of its kind to identify problems and gaps in terms of sociocultural determinants and mechanisms and policies to reduce salt, sugar, and fat in Iran. We could not convince a few interviewees to participate in our research despite our utmost efforts, perhaps due to their concern about their position. Nonetheless, the in-depth nature of the interviews with various stakeholders enabled us to collect a rich data source. Besides, this study was conducted before the COVID-19 pandemic initiated. We acknowledge that peoples’ diet may have changed due to economic consequences and food accessibility particularly among vulnerable citizens [36]. Our analysis is contextual-based and applicable to the characteristics of the Iranian society. Caution is necessary to generalize far-reaching conclusions from our study.

## Conclusions

This paper studied policymaking of the socio-cultural determinants in sugar, salt and fat consumption in Iran to help policymakers improve related policies. Reducing salt, fat and sugar consumption is problematic in Iran, mainly due to multi-dimensional socio-cultural determinants. In line with sustainable development goal (SDG) 3.4 to reduce 30% of premature death due to NCDs and related risk factors by 2030 in Iran, various stakeholders from multiple sectors, particularly the education system, media, civil society and government, need to initiate a coherent series of interventions to alter people’s approach to select healthy food, so they reduce the consumption of foods with excessive salt, fat and sugar.

## Supplementary Information

**Additional file 1.** Interview guide.

## Data Availability

The datasets used and/or analyzed during the current study available from the corresponding author on reasonable request. The entire dataset is in Farsi language.

## Abbreviations

NCDs: Noncommunicable diseases
NGOs: Non-Governmental Organizations.
SDG: Sustainable Development Goal
LMICs: Low and Middle-Income Countries
WHO: World Health Organization
WHA: The World Health Assembly
TUMS: The Tehran University of Medical Sciences
MA: Mohammad Amerzadeh
AT: Amirhossein Takian
MOHME: Ministry of Health and Medical Education
FDA: Food and Drug Administration
IRIB: Islamic Republic of Iran Broadcasting
HP: Hamed Pouraram
AKS: Ali Akbari Sari
AO: Afshin Ostovar
PhD: Philosophy of Doctrine
