# Supplementary material for: Policy analysis of socio-cultural determinants of salt, sugar and fat consumption in Iran
Source: BMC Nutr. 2022 Mar 25;8:26. doi: 10.1186/s40795-022-00518-7 (PMC8948451; doi:10.1186/s40795-022-00518-7)
Supplement: Supplementary file 1 — Additional file 1. Interview guide. [file 40795_2022_518_MOESM1_ESM.pdf]

Supplementary information:

Interview Guide:

I am Mohammad Amerzadeh, a PhD student in Health Policy, Tehran University of Medical Sciences, conducting research entitled “Identifying palatable policy options to decrease salt, sugar and fat consumption in Iran: An analysis for policy” under the supervision of Dr. Amirhossein Takian. We aim to study Iran’s sugar, fat and salt policies and compare them with other countries. Conducting this research requires knowledge of the policymakers and the health system managers and experts. Therefore, I request an interview with you to complete the information. Thank you so much for taking the time to answer these questions.

1. What policies are there in Iran to reduce the consumption of salt, sugar, and fat to prevent and control non-communicable diseases? Do you think these policies are a complete set, or is there a policy gap?

2. According to the recommendations of the World Health Organization, people should consume five units of fruit and vegetables a day. What policies do you think should be in place for people to consume more of these substances and consume less salt, sugar and fat? What are the obstacles?

3. What is your view on harmful goods that can lead to increased salt, sugar, and fat consumption in society? What legal gaps do you think there are?

4. What is the role of the private sector in reducing the consumption of salt, sugar and fat in Iran? What steps can they take to achieve this goal?

5 What is the role of the people and civil society in reducing the consumption of salt, sugar and fat in the country? What steps can they take to achieve this goal?

6. How do you think the industrial sector can reduce salt, sugar, and fat consumption in the country? Are these issues implemented in the country, and what are the problems?

7. What do you think of the food labelling process controlling sugar, salt, and fat? Is this way of implementing this policy appropriate now, or should it be changed?

8. How is the monitoring system related to food catering and their consumption in public organizations such as schools, universities and government centres, and restaurants to reduce the consumption of salt, sugar, and fat? What suggestions do you have for its better implementation?

9. Bead, cheese and Dough (Iranian drink made of yoghurt) are the primary source of salt in the country. Do you think these policies are enough? Do you think there is a specific policy that has not been implemented yet?

10. How feasible is the specific goal of zero trans-fatty acids by 2020 in " National Action Plan for the Prevention and Control of Non-Communicable Diseases and Related Risk Factors in the Islamic Republic of Iran, 2015-2025"? What are the problems?

11. What educational programs are implemented at the school level to reduce salt, sugar, and fat consumption? What other programs do you think should be implemented?

12. What role can media and social networks play in reducing salt, sugar and fat consumption in the country? Are these issues implemented in the country? What are the problems?

13. To what extent is the Iranian diet related to increasing the amount of salt, sugar and fat? What solutions do you suggest to reduce their consumption? Which one do you prioritize more?

14. What incentives and punishments are there to reduce these three components in the country? What other incentives and punishments do you consider appropriate?

15. What are the prominent key people that can reduce or increase the consumption of salt, sugar and fat in Iran? What can be done to improve their performance?

Finally, is there another point about reducing salt, sugar and fat consumption that needs to be discussed and has not been addressed?

Thank you for your time and attention.
